# Information Driven Self-Organization of Complex Robotic Behaviors

**DOI:** 10.1371/journal.pone.0063400

**Published:** 2013-05-27

**Authors:** Georg Martius, Ralf Der, Nihat Ay

**Affiliations:** 1 Max Planck Institute for Mathematics, Leipzig, Germany; 2 Santa Fe Institute, Santa Fe, New Mexico, United States of America; University of Vermont, United States of America

## Abstract

Information theory is a powerful tool to express principles to drive autonomous systems because it is domain invariant and allows for an intuitive interpretation. This paper studies the use of the predictive information (PI), also called excess entropy or effective measure complexity, of the sensorimotor process as a driving force to generate behavior. We study nonlinear and nonstationary systems and introduce the time-local predicting information (TiPI) which allows us to derive exact results together with explicit update rules for the parameters of the controller in the dynamical systems framework. In this way the information principle, formulated at the level of behavior, is translated to the dynamics of the synapses. We underpin our results with a number of case studies with high-dimensional robotic systems. We show the spontaneous cooperativity in a complex physical system with decentralized control. Moreover, a jointly controlled humanoid robot develops a high behavioral variety depending on its physics and the environment it is dynamically embedded into. The behavior can be decomposed into a succession of low-dimensional modes that increasingly explore the behavior space. This is a promising way to avoid the curse of dimensionality which hinders learning systems to scale well.

## Introduction

Autonomy is a puzzling phenomenon in nature and a major challenge in the world of artifacts. A key feature of autonomy in both natural and artificial systems is seen in the ability for independent exploration [1]. In animals and humans, the ability to modify its own pattern of activity is not only an indispensable trait for adaptation and survival in new situations, it also provides a learning system with novel information for improving its cognitive capabilities, and it is essential for development. Efficient exploration in high-dimensional spaces is a major challenge in building learning systems. The famous exploration-exploitation trade-off was extensively studied in the area of reinforcement learning [2]. In a Bayesian formulation this trade-off can be optimally solved [3], however it is computationally intractable. A more conceptual solution is to provide the agent with an intrinsic motivation [4], [5] for focusing on certain things and thus constraining the exploration to a smaller space. To approach this problem in a more fundamental way we consider mechanisms for goal-free exploration of the dynamical properties of a physical system, e. g. a robot. If the exploration is rooted in the agent in a self-determined way, i. e. as a deterministic function of internal state variables and not via a pseudo-random generator it has the chance to escape the curse of dimensionality. Why? Because specific features of the system such as constrains and other embodiment effects can be exploited to reduce the search space. Thus an exploration strategy taking the particular body and environment into account is vital for building efficient learning algorithms for high-dimensional robotic systems. But how can goal-free exploration be useful to actually pursue goals? We show that a variety of coordinated sensorimotor patterns are formed that may be used to quickly construct more complex behaviors using a second level of learning. It may also be used more directly in combination with reinforcement learning where the typical random exploration is substituted or augmented by the goal-free exploration leading presumably to a large speedup.

The solution for such a general problem needs a core paradigm in order to be relevant for a large class of systems. In recent years, information theory has come into the focus of researchers interested in a number of related issues ranging from quantifying and better understanding autonomous systems [6]–[12] to questions of spontaneity in biology and technical systems [13] to the self-organization of robot behavior [14], [15].

A systematic approach requires both a convenient definition of the information measure and a robust, real time algorithm for the maximization of that measure. This paper studies in detail the use of the predictive information (PI) of a robot’s sensorimotor process. The predictive information of a process quantifies the total information of past experience that can be used for predicting future events. Technically, it is defined as the mutual information between the past and the future of the time series. It has been argued [16] that predictive information, also termed excess entropy [17] and effective measure complexity [18], is the most natural complexity measure for time series. By definition, predictive information of the sensor process is high if the robot manages to produce a stream of sensor values with high information content (in the Shannon sense) by using actions that lead to predictable consequences. A robot maximizing PI therefore is expected to show a high variety of behavior without becoming chaotic or purely random. In this working regime, somewhere between order and chaos, the robot will explore its behavioral spectrum in a self-determined way in the sense discussed above.

This paper studies the control of robots by simple neural networks whose parameters (synaptic strengths and threshold values) are adapted on-line to maximize (a modified) PI of the sensor process. These rules define a mechanism for behavioral variability as a deterministic function formulated at the synaptic level. For linear systems a number of features of the PI maximization method have been demonstrated [14]. In particular, it could be shown that the principle makes the system to explore its behavior space in a systematic manner. In a specific case, the PI maximization caused the controller of a stochastic oscillator system to sweep through the space of available frequencies. More importantly, if the world is hosting a latent oscillation, the controller will learn by PI maximization to go into resonance with this inherent mode of the world. This is encouraging, since maximizing the PI means (at least in this simple example) to recognize and amplify the latent modes of the robotic system.

The present paper is devoted to the extension of the above mentioned method to nonlinear systems with nonstationary dynamics. This leads to a number of novel elements in the present approach. Commonly information theoretic measures are optimized in the stationary state. This is not adequate for a robot in a self-determined process of behavioral development. This paper develops a more appropriate measure for this purpose called the time-local predictive information (TiPI) for general nonstationary processes by using a specific windowing technique and conditioning. Moreover, the application of information theoretic measures in robotics is often restricted to the case of a finite state-action space with discrete actions and sensor values. Also these restrictions are overcome in this paper so that it can be used immediately in physical robots with high dimensional state-action space. This will be demonstrated by examples with two robots in a physically realistic simulation. The approach is seen to work from scratch, i. e. without any knowledge about the robot, so that everything has to be inferred from the sensor values alone. In contrast to the linear case the nonlinearities and the nonstationarity introduce a number of new phenomena, for instance the self-switching dynamics in a simple hysteresis system and the spontaneous cooperation of physically coupled systems. In high-dimensional systems we observe behavioral patterns of reduced dimensionality that are dependent on the body and the environment of the robot.

### Relation to Other Work

Finding general mechanisms that help robots and other systems to more autonomy, is the topic of intensive recent research. The approaches are widely scattered and follow many different routes so that we give in the following just a few examples.

#### Information theoretic measures

Information theory has been used recently in a number of approaches in robotics in order (i) to understand how input information is structured by the behavior [7], [19] and (ii) to quantify the nature of information flows inside the brain [8]–[10] and in behaving robots [11], [12]. An interesting information measure is the empowerment, quantifying the amount of Shannon information that an agent can “inject into” its sensor through the environment, affecting future actions and future perceptions. Recently, empowerment has been demonstrated to be a viable objective for the self-determined development of behavior in the pole balancer problem and other agents in continuous domains [20].

Driving exploration by maximizing PI can also be considered as an alternative to the principle of homeokinesis as introduced in [21], [22] that has been applied successfully to a large number of complex robotic systems, see [23]–[26] and the recent book [27]. Moreover, this principle has also been extended to form a basis for a guided self-organization of behavior [27], [28].

#### Intrinsic motivation

As mentioned above, the self-determined and self-directed exploration for embodied autonomous agents is closely related to many recent efforts to equip the robot with a motivation system producing internal reward signals for reinforcement learning in pre-specified tasks. Pioneering work has been done by Schmidhuber using the prediction progress as a reward signal in order to make the robot curious for new experiences [29]–[31]. Related ideas have been put forward in the so called play ground experiment [32], [33]. There have been also a few proposals to autonomously form a hierarchy of competencies using the prediction error of skill models [34] or more abstractly to balance skills and challenges [35]. Predictive information can also be used as an intrinsic motivation in reinforcement learning [36] or additional fitness in evolutionary robotics [37].

#### Embodiment

The past two decades in robotics have seen the emergence of a new trend of control in robotics which is rooted more deeply in the dynamical systems approach to robotics using continuous sensor and action variables. This approach yields more natural movements of the robots and allows to exploit embodiment effects in an effective way, see [38], [39] for an excellent survey. The approach described in the present paper is tightly coupled to the ideas of exploiting the embodiment, since the development of behavioral modes is entire dependent on the dynamical coupling of the body, brain, and its environment.

#### Spontaneity

We would like to briefly discuss the implications of using a self-determined and deterministic mechanism of exploration to the understanding of variability in animal behavior. Self-determined is understood here has “only based its own internal laws”. In the animal kingdom, there is increasing evidence showing that animals from invertebrates to fish, birds, and mammals are equipped with a surprising degree of variety in response to external stimulation [40]–[43]. So far, it is not clear how this behavioral variability is created. Ideas cover the whole range from the quantum effects [44] (pure and inexorable randomness) to thermal fluctuations at the molecular level to the assumption of pure spontaneity [45], rooting the variability in the existence of intrinsic, purely deterministic processes.

This paper shows that a pure spontaneity is enough to produce behavioral variations, and as in animals, their exact source appears “indecipherable” from an observer point of view. If the variation of behavior in animals is produced in a similar way, this would bring new insights into the free will conundrum [13].

## Methods

We start with the general expressions for the predictive information (PI) and introduce a derived quantity called time-local predictive information (TiPI) more suitable for the intended treatment of nonstationary systems. Based on the specific choice of the time windows we derive estimates of the TiPI for general stochastic dynamical systems and give explicit expressions for the special case of a Gaussian noise. The explicit expressions are used for the derivation of the parameter dynamics of the controller (exploration dynamics) obtained by gradient ascending the TiPI. Besides giving the exploration dynamics as a batch rule we also derive, in the sense of a stochastic gradient rule, the one-shot gradient. The resulting combined dynamics (system plus exploration dynamics) is a deterministic dynamical system, where the self-exploration of the system becomes a part of the strategy. These general results are then applied to the case of the sensorimotor loop and we discuss their Hebbian nature.

### Predictive Information

The PI of a time discrete process 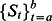 with values in 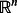 is defined [16] as the mutual information between the past and the future, relative to some instant of time 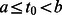


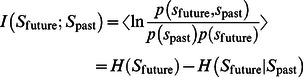
(1)where the averaging is over the joint probability density distribution 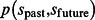 with 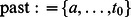 and 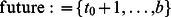. In more detail, we use the (differential) entropy 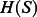 of a random variable 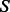 given by



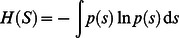
where 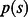 is the probability density distribution of the random variable 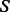. The conditional entropy 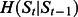 is defined accordingly



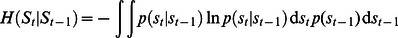


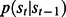
 being the conditional probability density distribution of 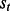 given 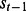. As is well known, in the case of continuous variables, the individual entropy components 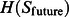, 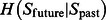 may well be negative whereas the PI is always positive and will exist even in cases where the individual entropies diverge. This is a very favorable property deriving from the explicit scale invariance of the PI [14].

The usefulness of the PI for the development of explorative behaviors of autonomous robots has been discussed earlier, see [14], [46], [47]. It was found in experiments with a coupled chain of wheeled robots [47] that the PI of just a single sensor, one of the wheel counters of an individual robot, already yields essential information on the behavior of the robot chain. The PI turned out to be maximal if the individual robots managed to cooperate so that the chain as a whole could navigate effectively. This is remarkable in that a one-dimensional sensor process can already give essential information on the behavior of a very complex physical object under real world conditions. These results give us some encouragement to study the role of PI and other information measures for specific sensor processes as is done in the present paper. This paper continues these investigations for the case of more general situations. In order to do so, we have to introduce some specifications necessary for the development of a versatile and stable algorithm realizing the increase of PI in the sensor process at least approximately.

Let us start with simplifying eq. (1). If 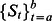 is a Markov process, see [46], the PI is given by the mutual information (MI) between two successive time steps, i. e. instead of eq. (1) we have
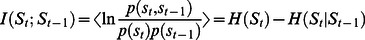
(2)the averaging being done over the joint probability density 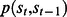. Actually, any realistic sensor process will only be in exceptional cases purely Markovian. However, we can use the simplified expression (2)–let us call it the one-step PI–also for general sensor processes taking it as the **definition** of the objective function driving the autonomous exploration dynamics to be derived.

#### Nonstationarity and Time-local Predictive Information (TiPI)

Most applications done so far were striving for the evaluation of the PI in a stationary state of the system. With our robotic applications, this is neither necessary nor adequate. The robot is to develop a variety of behavioral modes ideally in a open-ended fashion, which will certainly not lead to a stationary distribution of sensor values. The PI would change on the timescale of the behavior. How can one obtain in this case the probability distributions of 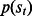
? The solution we suggest is to introduce a conditioning on an initial state in a moving time window and thus obtain the distributions from our local model as introduced below. More formally, let us consider the following setting. Let 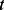 be the current instant of time and 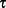 be the length of a time window 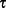 steps into the past. We study the process in that window with a fixed starting state 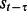 so that all distributions in eq. (2) are conditioned on state 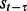. For instance, instead of 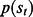 in eq. (2), we have to use
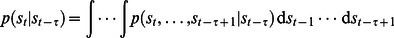
(3)and the related expression for 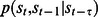, where 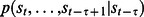 is the joint probability distribution for the process in the time window, conditioned on 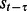. In the Markovian case eq. (3) boils down to 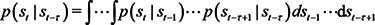. As to notation, the conditional probabilities depend explicitly on time so that 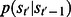 is different from 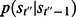 in general if 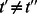, with equality only in the stationary state. As a result we obtain the new quantity, written in a short-hand notation as
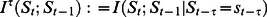
(4)which we call time-local predictive information (TiPI). Note the difference to the conditional mutual information where an averaging over 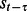 would take place. Analogously we define the time local entropy as 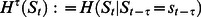
(5)


### Estimating the TiPI

To evaluate the TiPI only the kernels have to be known which can be sampled by the agent on the basis of the measured sensor values. However, in order to get explicit update rules driving the increase of the TiPI, these kernels have to be known as a function of the parameters of the system, in particular those of the controller. This can be done by learning the kernels as a function of the parameters. A related approach, followed in this paper, is to learn a model of the time series, i.e. learning a function 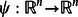 acting as a time series predictor 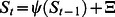 with realization
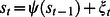
(6)for any time 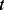, 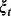 being the prediction error, also called the noise in the following. 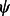 can be realized for instance by a neural network that can be trained with any of the standard supervised learning techniques. A concrete example will be considered below, see eq. (25). The relation to the kernel notation is obtained by observing that
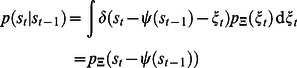
(7)where 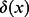 is the Dirac delta distribution and 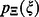 is the probability density of the random variable 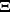 (prediction error) which may depend on the state 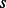 itself (multiplicative noise).

The case of linear systems, where 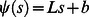 with a constant matrix 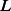, has been treated in [14] revealing many interesting properties of the PI. How can we translate the findings of the linear systems to the case of nonlinear systems? As it turns out, the nonlinearities introduce many difficulties into the evaluation of the PI as it becomes clear already in a one-dimensional bistable system as treated in [46]. Higher dimensional systems bring even more of such difficulties so that we propose to consider the information quantity on a new basis. The idea is to study the TiPI of the error propagation dynamics in the stochastic dynamical system instead of the process 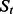 itself.

#### Error propagation dynamics

**Figure 1:**
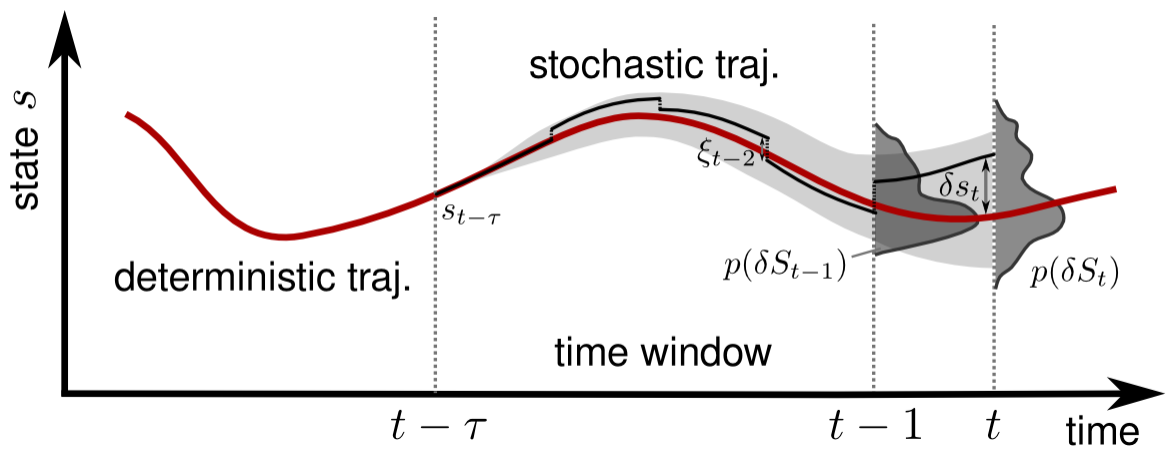
The time window and the error propagation dynamics used for calculating the TiPI, eq. (11). In principle, the process is considered many times with always the same starting value but different realizations of the noise 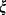. Note that, when using the one-shot gradients, only one realization is needed.



Let us introduce a new variable describing the deviation of the actual dynamics, eq. (6), from the deterministic prediction in a certain time window. We define for a time window starting at time 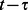


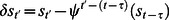
(8)for any time 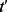 with 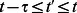 and 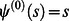. As to notation, 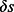 denotes a single variable not to be confused with the Dirac function. Intuitively 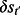 captures how the prediction errors occurred since the start of the time window are propagated up to time 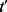. Figure 1 illustrates the transformed state and the relevant distributions of the belonging process 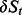. Interestingly the TiPI on the process 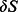 is equivalent to the one on the original process 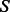, see eq. (A5) in Text S1. The dynamics of the 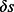 can be approximated by linearization as

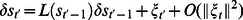
(9)using the Jacobian



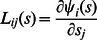
(10)Assuming the prediction errors (noise) 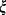 to be both small and Gaussian we obtain an explicit expression for the TiPI on 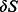


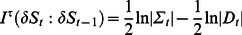
(11)where 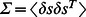 is the covariance matrix of 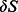 and 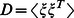 is the covariance matrix of the noise. The derivation and further details are in section A in Text S1. The results for linear systems in [14] can be obtained from the general case considered here by 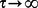.

When looking at eq. (11) one sees that the entropies are expressed in terms of covariance matrices. This is exact in the case of Gaussian distributions. In the general case this may be considered as an approximation to the true TiPI. Alternatively, we can also consider eq. (11) as the definition of a new objective function for any process if we agree to measure variability not in terms of entropies but more directly in terms of the covariance matrices.

### The Exploration Dynamics

Our aim is the derivation of an algorithm driving the behavior of the agent toward increasing TiPI. Let us assume that the function 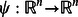 depends on a set of parameters 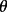 so that we may write the dynamics as
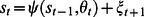
(12)


For instance, if 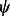 is a neural network as introduced further below, the parameter set 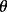 comprises just the synaptic weights and threshold values of the neurons.

#### Gradient ascending the TiPI

Based on the TiPI, eq. (11), a rule for the parameter dynamics is given by the gradient step to be executed at each time 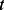


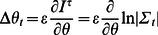
(13)where 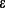 is the update rate and 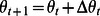. The term 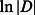 from eq. (11) has been omitted assuming that 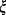 is essentially noise which is not depending on the parameters of the controller. This is justifiable in the case of parsimonious control as realized by the low-complexity controller networks. These generate typically well predictable (low noise) behaviors as shown in the applications studied below.

In order to get more explicit expressions, let us consider the case of very short time windows. With 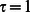 there is no learning signal since 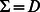 meaning that 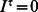. So, 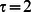 is the most simple nontrivial case. The parameter dynamics is given by
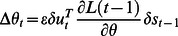
(14)where 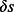 and the auxiliary vector 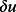 are given as



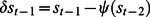
(15)

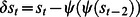
(16)

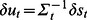
(17)

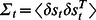
(18)stipulating the noise is different from zero (though possibly infinitesimal) and employing the self-averaging property of a stochastic gradient, see below. The general parameter dynamics for arbitrary 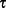 is derived in section B in Text S1. However, in the applications described below, already the simple parameter dynamics with 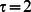 will be seen to create most complex behaviors of the considered physical robots.

In a nutshell, eq. (13) reveals already the main effect of TiPI maximization: increasing 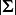 means increasing the norm of 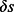 (in the 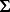-metric see eq. (A21) in Text S1). This is achieved by increasing the amplification of small fluctuations in the sensorimotor dynamics which is equivalent to increasing the instability of the system dynamics, see also the more elaborate discussion in [47].

#### Learning vs. exploration dynamics

Usually, updating the parameters of a system according to a given objective is called learning. In that sense, the gradient ascent on the TiPI defines a learning dynamics. However, we would like to avoid this notion here, since actually nothing is learnt. Instead by the interplay between the system and the parameter dynamics, the combined system never reaches a final behavior corresponding to the goal of a learning process. Therefore we prefer the notion *exploration dynamics* for the dynamics in the parameter space that is driven by the TiPI maximization.

#### One-shot gradients

The formulas for the gradient (eqn. (14) and (A21) in Text S1) were obtained by tacitly invoking the self-averaging properties of the gradient, i. e. by simply replacing 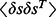 with 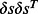 in eq. (A16) in Text S1. This still needs a little discussion. Actually, the self-averaging is exactly valid only in the limit of sufficiently small 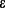, with 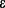 eventually being driven to zero in a convenient way. However, our scenario is different. What we are aiming at is the derivation of an intrinsic mechanism for the self-determined and self-directed exploration using the TiPI and related objectives. The essential point is that self-exploration is driven by a deterministic function of the states (sensor values) of the system itself.

Equation (14) obtained from the gradient of the TiPI fulfills these aims very well–any change of the system parameters and hence of the behavior is given in terms of the predecessor states in the short time window. With finite (and often quite large) 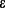 eqs. (14)–(18) are just a rough approximation of the original TiPI but, in view of our goal, the one-shot nature of the gradient is favorable as it supports the explorative nature of the exploration dynamics generating interesting synergy effects.

#### Synergy of system and exploration dynamics

A further central aspect of our approach is the interplay between the system and the parameter dynamics driven by the TiPI maximization process. In specific cases, the latter may show convergence as in conventional approaches based on stationary states. An example is given by the one-parameter system studied in [46] realizing convergence to the so called effective bifurcation point. However, with a richer parametrization and/or more complex systems, instead of convergence, the combined system (state+parameter dynamics) never comes to a steady state due to the intensive interplay between the two dynamical components if 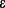 is kept finite. An example will be given in the Results section.

Typically, the TiPI landscape permanently changes its shape due to the fact that increasing the TiPI means in general a destabilization of the system dynamics. If the latter is in an attractor, increasing the TiPI destabilizes the attractor until it may disappear altogether with a complete restructuring of the TiPI landscape. This is but one of the possible scenarios where the exploration dynamics engages into an intensive and persistent interplay with the system dynamics. This interplay leads to many synergistic effects between system and exploration dynamics and makes the actual flavor of the method.

#### Self-directed search

The common approach to solve the exploration–exploitation dilemma in learning problems is to use some randomization of actions in order to get the necessary exploration and then decrease the randomness to exploit the skills acquired so far. This is prone to the curse of dimensionality if the systems are gaining some complexity. Randomness can also be introduced by using a deterministic policy with a random component in the parameters, as quite successfully applied to evolution strategies and reinforcement learning [48], [49].

Our approach is also to use deterministic policies (given by the function 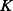) but aims at making exploration part of the policy. So, instead of relegating exploration to the obscure activities of a random number generator, variation of actions should be generated by the responses of the system itself. This replaces randomness with spontaneity and is hoped (and will be demonstrated) to restrict the search space automatically to the physically relevant dimensions defined by the embodiment of the system.

Formally, we call a search self-directed if there exists a function 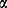 so that the change in the parameters
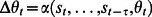
(19)is given as a deterministic function of the states in a certain time window (of length 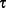) and the parameter set 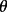 itself. In this paper, 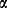 is given by the gradient of the predictive information in the one-shot formulation.

In more general terms, we believe that randomization of actions makes the agent heteronomous, its fate being determined by an obscure (to him) procedure (the pseudo-random number generator) alien to the nature of its dynamics. The agent is autonomous in the ‘genuine’ sense only if it varies its actions exclusively by its own internal laws [50]. In our approach, according to eq. (19), exploration is driven entirely by the dynamics of the system itself so that exploration is coupled in an intimate way to the pattern of behavior the robot is currently in. The danger might be that in this way the exploration is restricted too much. As our experiments show, this is not so for active motion patterns in high dimensional systems. This fact can be attributed to the destabilization effect incurred by the TiPI maximization, see above and [47]. For stabilizing behaviors, however, the exploration may be too restrictive.

### The Sensorimotor Loop

Let us now specify the above expressions to the case of a sensorimotor loop, in particular a neurally controlled robotic system. The dynamical systems formulation is obtained now by writing our predictor for the next sensor values as a function of both the sensors and the actions so that
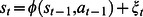
(20)where 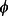 represents the so-called forward model and 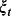 is the prediction error as before. As the next step, we consider the controller also as a deterministic function 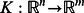 generating actions (motor values) 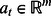 as a function of the sensor values 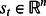 so that



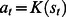
(21)In the applications, 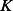 will be realized as a (feed-forward) neural network. Using eq. (21) in eq. (20) we obtain the map 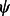 modeling our sensor process as
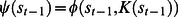
(22)


In [14] a standard linear control system was studied where 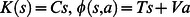 and 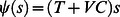. This paper will consider a nonlinear generalization of that case in specific robotic applications.

#### Exploration dynamics for neural control systems

In the present setting, we assume that both the controller 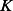 and the forward model 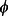 of our robot are realized by neural networks, the controller being given by a single-layer neural network as
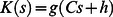
(23)the set of parameters 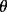 now given by 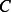 and 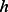. In the concrete applications to be given below, we specifically use 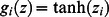 (to be understood as a vector function so that 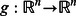)

Moreover, the forward model 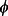 is given by a layer of linear neurons, so that
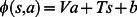
(24)


The matrices 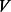, 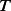 and the vector 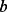 represent the parametrization of the forward model that is adapted on-line by a supervised gradient procedure to minimize the prediction error 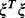 as
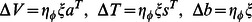
(25)


In the applications, the learning rate 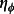 is large such that the low complexity of the model is compensated by a very fast adaptation process.

In contrast to the forward model parameters, the controller parameters are to be adapted to maximize the TiPI. For that the map 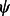 (eq. (22)) is required which becomes 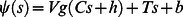 with Jacobian matrix
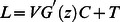
(26)where 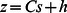 is the postsynaptic potential and
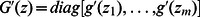
(27)is the diagonal matrix of the derivatives of the activation functions for each control neuron.

In the applications given below, we are using the short-time window, with the general exploration dynamics given by eq. (14). The explicit exploration dynamics for this neural setting with 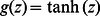 are given as
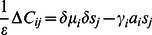
(28)

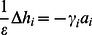
(29)where all variables are time dependent and are at time 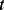, except 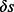 which is at time 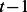. The vector 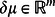 is defined as
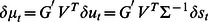
(30)(see eq. (17)), and the channel specific learning rates 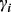 are



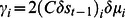
(31)The derivation and generalization to aribrary activation functions are provided in section C in Text S1. The update rules for 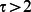 are given by a sum of such terms, with appropriate redefinitions of the vector 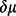, see eq. (A20) in Text S1.

#### The Hebbian nature of the update rules

In order to interpret these rules in more neural terms, we at first note that the last term in eq. (28) is of an anti-Hebbian structure. In fact, it is given by the product of the output value 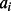 of neuron 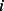 times the input 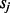 into the 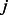-th synapse of that neuron, the 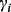 (which are positive, as a rule) being interpreted as a neuron specific learning rate. Moreover, we may also consider the term 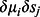 as a kind of Hebbian since it is again given by a product of values that are present at the ports of the synapse 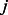 of neuron 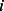. The factor 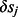 can be considered as a signal directly feeding into the input side of the synapse 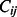. Moreover, 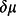 given as 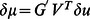 is obtained by using 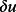 as the vector of output errors in the 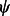 network and propagating this error back to the layer of the motor neurons by means of the standard backpropagation algorithm.

These results make the generalization to more complicated, multi-layer networks straightforward. However already the simple setting produces an overwhelming behavioral variety, see the case studies below.

More intuitively the Hebbian term acts as a self-amplification and increases the Lyapunov exponents. In the linear case [14] this leads eventually to the divergence of the dynamics such that the PI does not exist any longer. With the nonlinearities, the latter effect is avoided, but the system is driven into the saturation region of the motor neurons. However, the second term in eq. (28), by its anti-Hebbian nature, is seen to counteract this tendency. The net effect of both terms is to drive the motor neurons towards a working regime where the reaction of the motors to the changes in sensor values is maximal. This is understandable, given that maximum entropy in the sensor values requires a high sensorial variety that can be achieved by that strategy.

## Results

We apply our theory to three case studies to illuminate the main features. First a hysteresis systems is considered to exemplify the consequences of nonstationarity and the resulting interplay between the exploration dynamics and the system dynamics in a nutshell. In section “Spontaneous cooperation with decentralized control” a physical system of many degrees of freedom is controlled by independent controllers that spontaneously cooperate. Finally in section “High dimensional case – the Humanoid ” we apply the method to a jointly controlled humanoid robot in various situations to illustrate the exploration process in a high-dimensional embodied system.

### Hysteresis Systems

Nonstationary processes are the main target of our theory, made accessible by the special windowing and averaging technique presented in this paper for the first time. In order to work out the consequences, let us consider an idealized situation where the above derivations, in particular eqs. (14)–(18), are the exact update rules for increasing the TiPI.

Let us consider a single neuron in an idealized sensorimotor loop, where the sensor values are 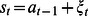 (the white Gaussian noise 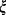 is added explicitly). This case corresponds to the dynamical system
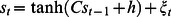
(32)where now 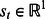. The system was studied earlier [47] in the special case of 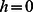 and it was shown that the maximization of the PI self-regulates the system parameter 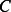 towards a slightly supercritical value 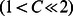. There, the system is at the so called effective bifurcation point where it is bistable but still sensitive to the noise.

**Figure 2:**
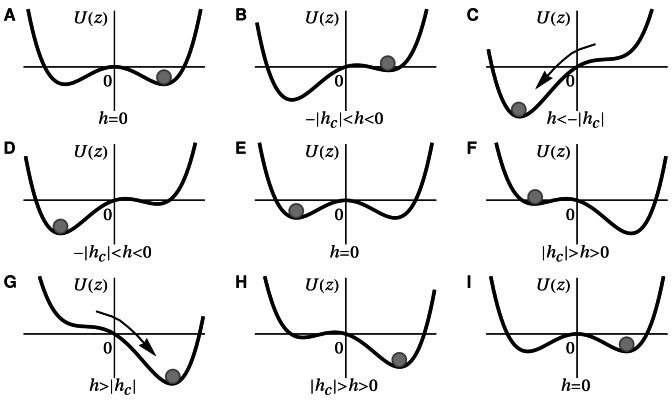
The hysteresis cycle in the gradient picture. The diagrams show the stages of one hysteresis cycle starting from 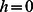 (**A**) with the state at 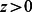 as represented by the sphere. Decreasing 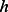 creates the asymmetric situation (**B**). If 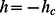 the saddle-node bifurcation happens, i. e. both the maximum at z = 0 and the right minimum disappear so that the system shifts to the left minimum of the potential (**C**). Increasing 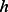 until 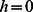 brings us back to the initial situation with the state shifted to the other well see (**D,E**). The diagrams (**F**) and (**G**) depict the switching from the minimum at 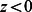 to the minimum at 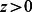 by increasing 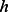. By decreasing 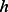 until 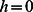 the hysteresis cycle is finished, see (**H,I**).

Let us start with keeping 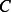 fixed at some supercritical value (e. g. 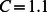) and concentrating on the behavior of the bistable system as a function of the threshold value 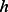. The interesting point is that the system shows hysteresis. This can be demonstrated best by rewriting the dynamics in state space as a gradient descent. Let us introduce the postsynaptic potential 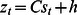 and rewrite eq. (32) in terms of 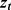 as
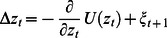
(33)where 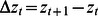 and the potential is 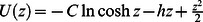 (using 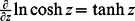). In that picture, the hysteresis properties of the system are most easily demonstrated by Fig. 2. This phenomenon can be related directly to the destabilization effect of the exploration dynamics. In the potential picture, stability is increasing with the well depth. Hence, the exploration dynamics, aiming at the destabilization of the system, is decreasing the depth of the well more and more until the well disappears altogether, see Fig. 2, and the state switches to the other well where the procedure restarts.

#### Deterministic self-induced hysteresis oscillation

Now we show that in the one-dimensional case the parameter dynamics is independent of white noise. This implies we can in the state dynamics make the limit of vanishing noise strength and obtain a fully deterministic system. Again we only consider the two-step window (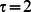). Using 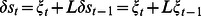 (eq. (9)) we find that the TiPI, according to eq. (A15) in Text S1

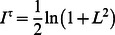
is independent of the noise. Analogously to eqs. (28)–(31) we obtain the update rules for 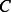 and 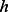 as the gradient ascent on 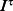 and thus the full state-parameter dynamics (with 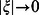) is given by



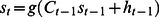
(34)

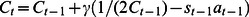
(35)

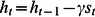
(36)with 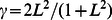.

**Figure 3:**
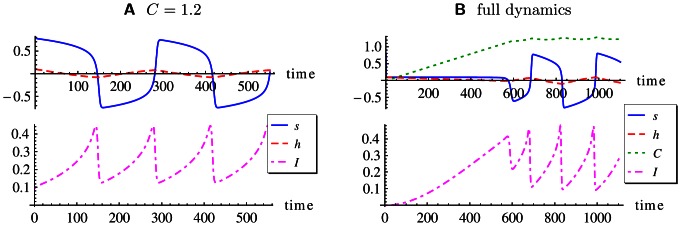
State and parameter dynamics in the one-dimensional system. (**A**) Only 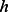 dynamics (fixed 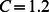); the bias 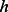 oscillates around zero and causes the state 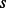 to jump between the positive and negative fixed points. The TiPI is seen to increase steadily until it eventually drops back when the state is jumping. (**B**) With full dynamics (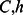). 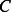 increases until it oscillates around its average at 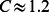 where the hysteresis cycle starts. Parameters: 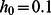, 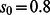, 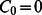 in (**B**), 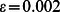.

Apart from the definition of 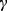 (that just modulates the speed of the parameter dynamics), the extended dynamical system agrees in the one-dimensional case with that derived from the principle of homeokinesis, discussed in detail in [27]. Let us therefore only briefly sketch the most salient features of the dynamics. Keeping 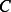 fixed at some supercritical value, as above the most important point is that, instead of converging towards a state of maximum TiPI, the 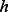 dynamics drives the neuron through its hysteresis cycle as shown in Fig. 2, which we call a self-induced hysteresis oscillation, see Fig. 3 (A).

For the full dynamics (with eq. (35)) the results are given in Fig. 3 (B) showing that the feedback strength 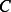 in the loop converges indeed toward the regime with the hysteresis oscillation. This demonstrates that the latter is not an artifact present only under the specific parametrization. In fact, we encounter this phenomenon in many applications with complex high-dimensional robotic systems, see the experiments with the Armband below and many examples treated in [27].

Interestingly this behavior is not restricted to simple hysteresis systems but is of more general relevance. For instance, in two-dimensional systems a second order hysteresis was observed, corresponding to a sweep through the frequency space of the self-induced oscillations [27]. It would be interesting to relate this fast synaptic dynamics to the spike-timing-dependent plasticity [51] or other plasticity rules [52] found in the brain.

#### About time windows

**Figure 4:**
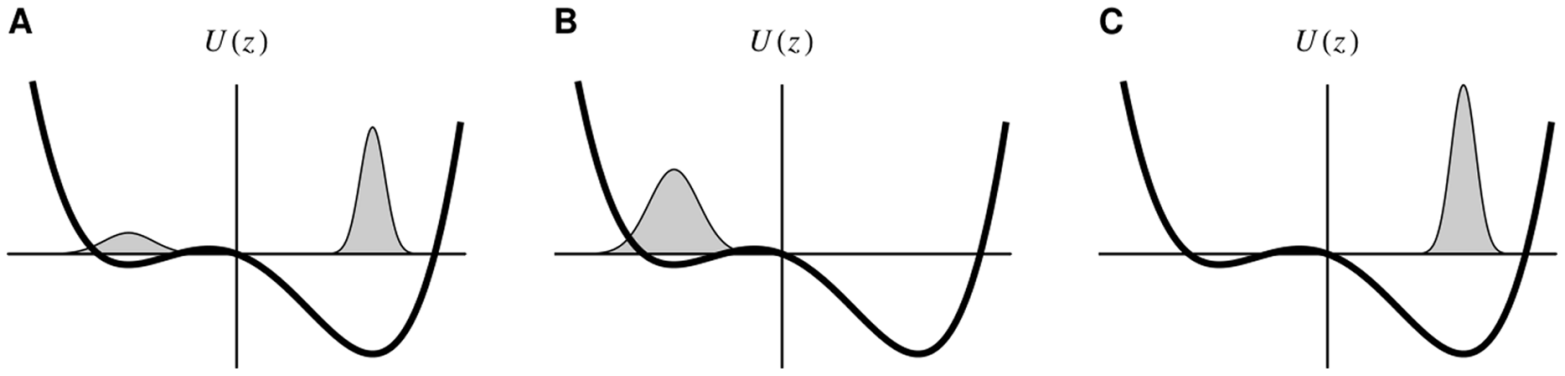
The probability density distributions with different time windows of the stochastic process in an asymmetric double well potential. The mean first passage time 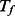 of switching between wells is one characteristic time constant of the process [53], 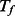 increasing exponentially with the barrier height. If observing the process in a window of length 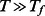, the distribution of (**A**) will be observed. In that situation, the TiPI is maximal if the wells are of equal depth (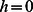). However, with windows of length 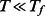, the system state will be predominantly in one of the wells generating the distributions shown in (**B**), (**C**). Gradient ascending the TiPI will decrease the well depth as long as the probability mass is still concentrated in that well. This is what drives the hysteresis cycle depicted in Fig. 2.

Before giving the applications to embodied systems, let us have a few remarks on the special nature of the time windowing technique as compared to the common settings. Let us consider again the bistable system with the bias 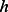 as the only parameter and with finite noise. Figure 4 depicts a typical situation with 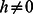 so that the wells are of different depth. The figure depicts the qualitative difference between the classical attitude of considering information measures in very large time windows, large enough for the process to reach total equilibrium, as compared to our nonstationarity approach where the TiPI is estimated on the basis of a comparatively short window. Note that the time to stay in a well is exponentially increasing with the depth of the well and decreasing exponentially with the strength of the noise [53]. Mean first passage times can readily exceed physical times (on the time scale of the behavior) by orders of magnitudes.

While in the former case convergence of the hysteresis parameter 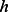 towards the equilibrium condition 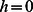 is reached, there is no convergence in the nonstationary case. Instead, one obtains a self-induced hysteresis oscillation. This is generic for a large class of phenomena based on the synergy effects between system and exploration dynamics which open new horizons for the explorative capabilities of the agent. In the context of homeokinesis, this phenomenon has already been investigated in many applications, see [27]. This paper provides a new, information theoretic basis and opens new horizons for applications as the matrix inversions inherent to the homeokinesis approach are avoided.

### Spontaneous Cooperation with Decentralized Control

Let us now give examples illustrating the specific properties of the present approach. We start with an example of strongly decentralized control where the TiPI driven parameter dynamics leads to the emergence of collective modes. Earlier papers have already demonstrated this phenomenon for a chain of passively coupled mobile robots [15], [46], [47]. In the setting of [46], [47], each wheel was being controlled by a single neuron with a synapse of strength 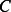 defining the feedback strength in each of the sensorimotor loops. There was no bias. As it turned out, the TiPI in the sensorimotor loop is maximal if the synaptic strength 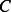 is at its critical value where the system is bistable but still reacts to the external perturbations, i. e. the system is at its so-called effective bifurcation point [27]. As compared to the present setting, these results correspond to using a time window of infinite length, stipulating the presence of a stationary state.

The situation is entirely different when using the short time window and large update rates allowing for the synergy effects. In experiments with the robot chain, we observe better cooperativity with the hysteresis oscillations and better exploration capabilities. The reason can be seen in the fact that the self-regulated bias oscillations help the chain to better get out of impasse situations. We do not give details here, since we will study in the following an example that demonstrates the synergy effects even more convincingly.

#### The Armband

**Figure 5:**
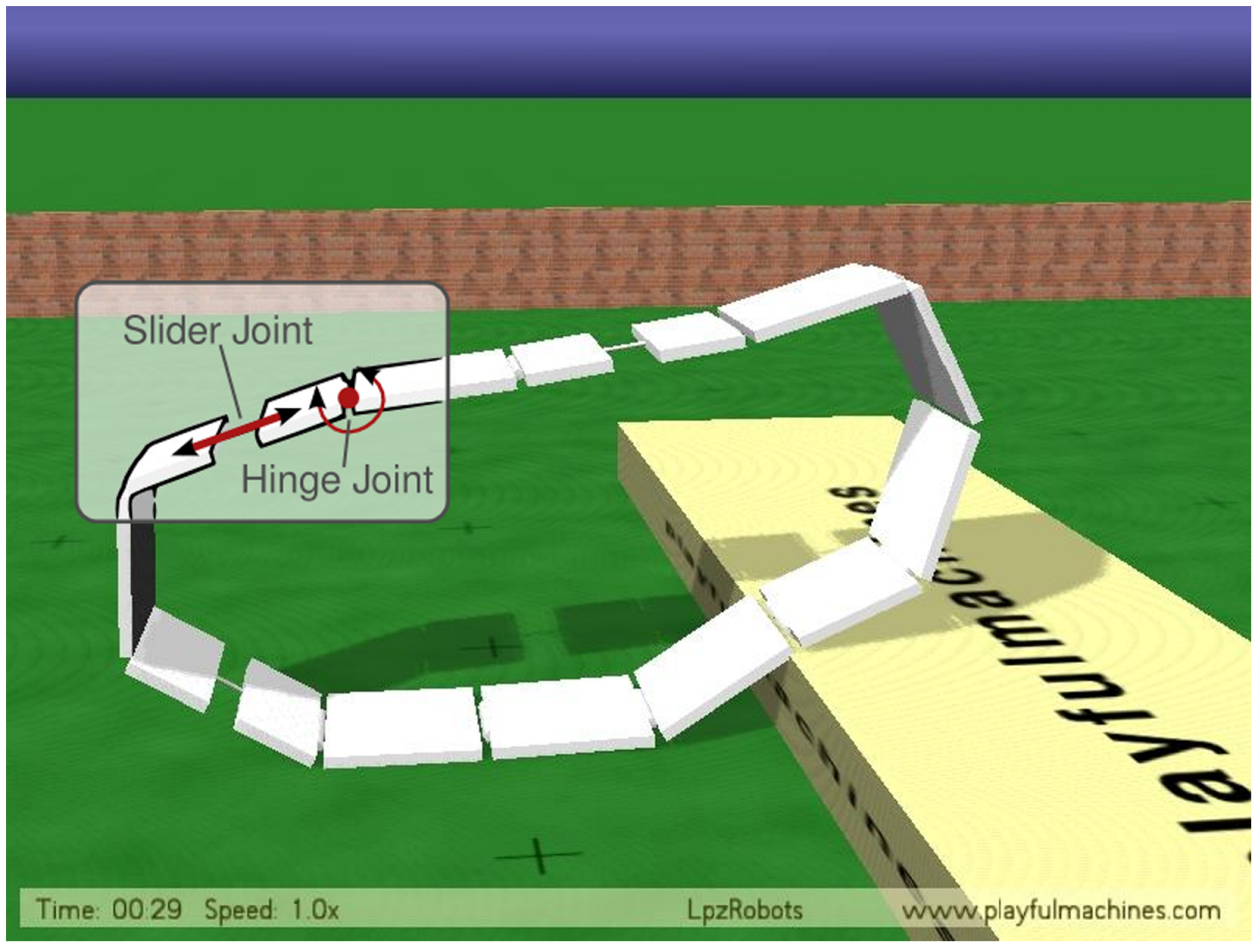
The Armband. The robot has 12 hinge and 6 slider joints, each actuated by a servo motor and equipped with a proprioceptive sensor measuring the joint angle or slider length. The robot is strongly underactuated so that it can not take on a wheel like form where locomotion were trivial.

The Armband considered here is a complicated physical object with 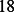 degrees of freedom, see Fig. 5. The physics of the robot is simulated realistically in the LpzRobots simulator [54]. The program source code for this and the next simulation is available from [55]. Each joint is controlled by an individual controller, a single neuron driven by TiPI maximization, as with the robot chain treated in [47]. The controller receives the measured joint angle or slider position and the output of the controller defines the target joint angle or target slider position to be realized by the motor. The motors are implemented as simulated servomotors in order to be as close to reality as possible. Moreover, the forces are limited so that, due to the interaction with obstacles or the entanglement of the system’s different degrees of freedom, the true joint angle may differ substantially from the target angle. These deviations drive the interplay between system and exploration dynamics.

In the experiments, we use the controller given by eq. (23) and the update rules for the parameter dynamics as given by eqs. (35) and (36). The adaptive forward model is given by eq. (24) with 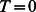 and the appropriate learning rules eq. (25). In order to demonstrate the constitutive role of the synergy effect, we started by studying the system with fixed 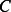 and 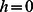. In contrast to the chain of mobile robots, with fixed parameters there is no parameter regime where the Armband shows substantial locomotion. This result suggests that, as compared to the chain of mobile robots, the specific embodiment of the Armband is more demanding for the emergence of the collective effect.

In order to assess the effects appropriately, note that potential locomotion depends on the forces the motors are able to realize. For instance, if the robot is strongly actuated, the command 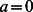 for each of the motors drives each joint to its center position so that the shape of the robot is nearly circular, locomotion readily taking place under the influence of very weak external influences. In order to avoid such trivial effects, we use an underactuated setting so that gravitational or environmental forces are deforming the robot substantially, see Fig. 5.

**Figure 6:**
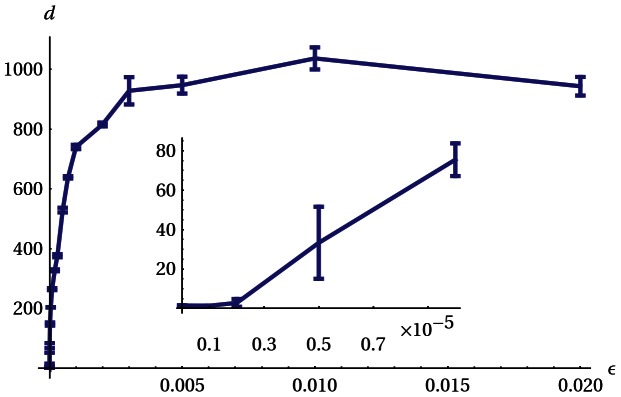
Role of the fast synaptic dynamics: depending on the speed of the synaptic dynamics defined by 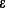, the locomotion properties are changing drastically. Depicted is the distance traveled by the robot in 10 min simulated time on an empty plane. The inset gives a close up view for low 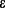, demonstrating that the locomotion starts only if 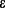 exceeds a certain threshold value. Shown is the mean and standard deviation of 10 runs each. Update frequency 25 Hz.

**Figure 7:**
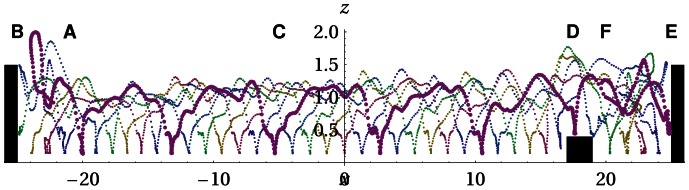
Regular locomotion pattern and interaction with the environment. Plotted are the center positions of the 6 rigid segments in space for an interval of 40 sec. One line is highlighted for visibility. The trajectory starts while the robot is moving to the left (**A**) and is hitting the wall (**B**) (black box) and locomotes to the right (**C**) showing a very regular pattern. Then it overcomes an obstacle (**D**) and hits the wall (**E**) and moves back (**F**). The behavior is cyclic. Parameter: 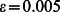.

**Figure 8:**
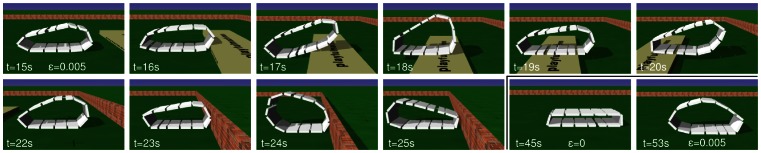
Armband robot surmounting an obstacle and inverting speed at a wall. Screen shots from the simulation for Fig. 7. The order is row-wise from left to right. The last two pictures show the situation after switching off the parameter dynamics 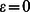 for a few seconds (the robots stops) and enabling it again (starts moving).

The situation changes drastically if the 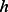 dynamics is included. As demonstrated by Fig. 6, substantial locomotion sets in only if 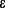 is large enough so that the exploration dynamics is sufficiently fast for the synergy effect to unfold. Also, as the experiments show, the effect is stable for a very wide range of 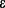 and under varying external conditions. It is also notable, that the Armband robot shows a definite reaction to external influences. For instance, obstacles in its path are either surmounted or cause the robot to invert its velocity, see Fig. 7. The latter effect is observed in particular in the underactuated regime defined above, so that the reflection is not the result of the elastic collision but it is actively controlled by the involvement of the exploration dynamics. The role of the latter is also demonstrated by the fact that locomotion stops as soon as the update rate 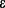 is put to zero, see Fig. 8 and the corresponding video S1.

The Armband has also been investigated recently using artificial evolution for the controller [56], demonstrating convincingly the usefulness of the evolution strategy for obtaining recurrent neural networks that make the Armband roll into a given direction. There are several differences to our approach, both conceptually and in the results. While in the evolution strategy the fitness function was designed for the specific task and many generations were necessary to get the performance, in our approach the rolling modes are emerging right away by themselves. Moreover, the modes are sensitive to the environment, for instance by inverting velocity upon collisions with a wall, they are flexible (changing to a jumping behavior on several occasions) and resilient under widely differing physical conditions. Interestingly, these behaviors are achieved with an extremely simple neural controller, the functionality of a recurrent network being substituted by the fast synaptic dynamics.

### High Dimensional case – the Humanoid

Let us now study the properties of the exploration dynamics in a general (not decentralized) control task. We consider a humanoid robot with 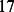 degrees of freedom. Each joint is driven by a simulated servo motor, the motor values 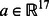 sent by the controller are the target angles of the joints and sensor values 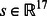 are the true, observed angles. This is the only knowledge the robot has about its physical state.

The aim of this experiment is to investigate in how far the robot develops behaviors with high variability so that it explores its sensorimotor contingencies. Given that there is no externally defined goal for the behavior development, will the robot develop a high behavioral variety depending on its physics and the environment it is dynamically embedded into?

**Figure 9:**
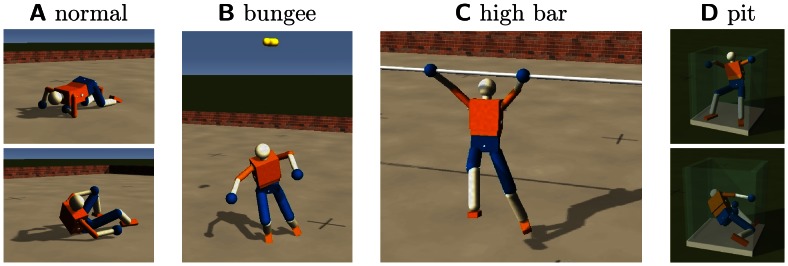
The Humanoid robot in four different scenarios. (**A**) Normal environment with flat ground. (**B**) The robot is hanging at a bungee like spring. (**C**) The robot is attached to a high bar. (**D**) Robot is fallen into a narrow pit.

**Figure 10:**
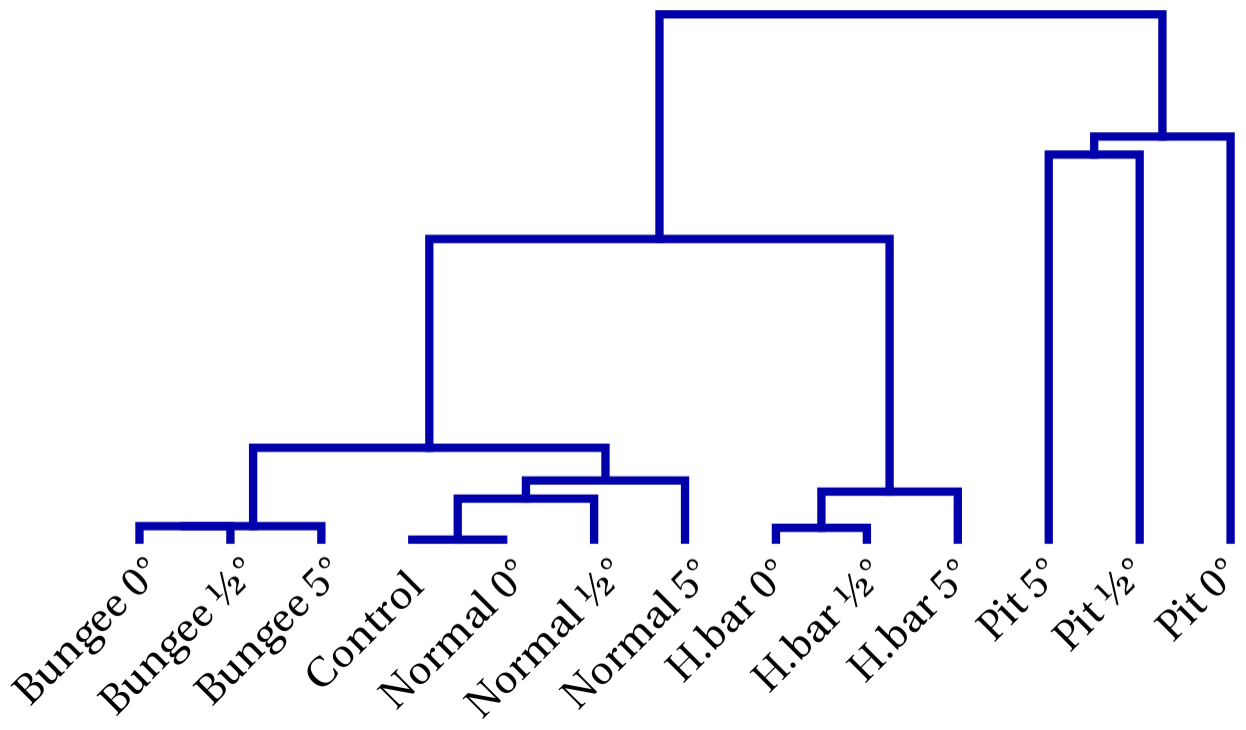
Parameter similarity for the behavior in different environments (Fig. **9).** Plotted is the results of a hierarchical clustering based on the difference between the parameters in each of the simulations (averaged over time). For each of the four environments there are three initial poses: 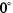 (straight upright), 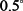 and 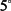 slanted to the front. The parameters for runs in the same environment are clustered together. This supports the observation that the embodiment plays an essential role in the generation of behavior. More importantly the physical conditions are reflected in the parameters and are thus internalized. We used the squared norm of the difference of the absolute values of the matrix elements. The absolute values were used because a common structure in the parameters are rotation matrices and there the same qualitative behavior is obtained with inverted signs. Parameters: 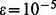, 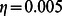, update frequency 50 Hz.

That this happens indeed is demonstrated by the videos S2, S3, S4 and S5. However, we want a more objective quantity to assess the relation between body and behavior. We provide two different measures for that purpose. One idea is to use the parameter constellation of the controller itself for characterizing the behavior–different behaviors should reflect in characteristic parameter configurations of the controller. In order to study this idea, we place the robot in different scenarios, see Fig. 9, always starting with the same initial parameter configuration (using the result of a preparatory learning phase in the bungee setting), letting the robot move independently for 40 min physical time. Without any additional noise, the dynamics is deterministic so that variations are introduced by starting the robot in different poses, i. e. in a straight upright position and in slightly tilted poses (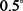 and 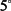 slanted to the front). We then compared the parameter values of the controller matrix 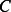 at each second (1 s) for all simulations and calculated a hierarchical clustering reflecting the differences between the 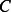 matrices. Figure 10 shows the resulting dendrogram.

Obviously, there is a distinct grouping of the 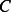 matrices according to the environment the robot is in and the behaviors developing in the respective situation. Distances between the groups are different, the most pronounced group corresponding to the behavior in the pit situation. This seems plausible since the constraints are most distinctive here, driving the robot to behaviors that are markedly different from the situation with the bungee setting, say, where all joints (extremities, hip, back) can move much more freely. There is a second pronounced group–the robot clinging to the high bar–whereas the distances between the 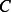 matrices controlling the robot lying on the ground and hanging at the bungee rope is less pronounced. However, by visual inspection the emerging behaviors in the two latter situations appear quite different (compare videos S2 and S3)–a finding that is not so clear in the matrix distance method.

In order to get an additional measure we start from the idea that the TiPI maximization method produces a series of behaviors that are qualified by a high dynamical complexity generated in a controlled way. The latter point means that the dimensionality of the time series of the sensor values is much less than that of the mechanical system – if the behavior of the robot is well controlled (think of a walking pattern) a few master observables will be sufficient to describe the dynamics of the mechanical system. We have tried different methods from dynamical system theory for finding the effective dimension of that time series without much success. The reason was found to be in the strongly nonstationary nature of the compound dynamics (system plus exploration dynamics) making low dimensional behaviors to emerge and disappear in a rapid sequence. So, in the long run the full space of the dynamical system is visited so that globally a seemingly high dimensional behavior is observed.

In order to cope with this nonstationary characteristic, we developed a different method, splitting the whole time series into chunks and using an elementary principal component analysis (PCA) in order to define the effective dimension in each chunk: on each chunk a PCA is performed and the number of principal components required to capture 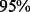 of the data’s variance is plotted (mean and standard deviation for all chunks of the same length). In order to avoid discretization artifacts we linearly interpolate the required number of components to obtain a real number.

**Figure 11:**
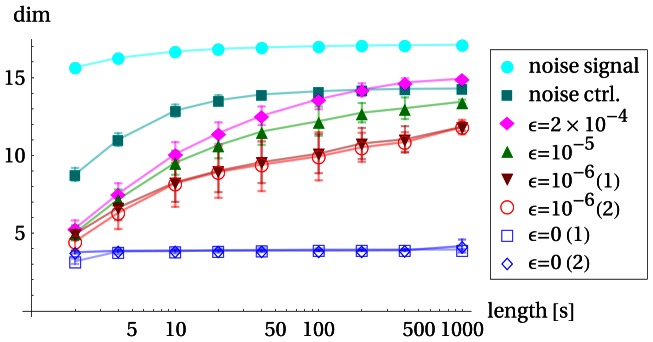
Dimensionality of behavior on different time scales. Humanoid robot in bungee setup running 40 min with different control settings. The sensor data is partitioned into chunks of a fixed length, the graph depicting the effective dimension over the length of the chunks for different settings. In order to test the method we start with a uniformly distributed noise signal for motor commands (“noise signal”). As expected the observed dimension is maximal. The sensor values produced by that random controller show a lower dimension (“noise ctrl.”) as is expected due to the low pass filtering property of the mechanical system. All other cases are with the TiPI maximization controller with different update rates 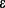. In particular, the comparison with the 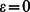 case demonstrates that the exploration dynamics produces more complex behaviors than any fixed controller.

**Figure 12:**
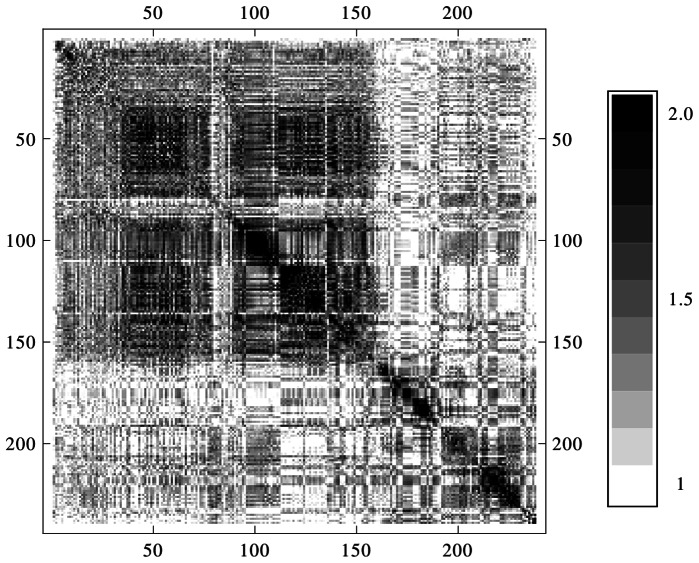
Behavioral changes with time. Pairwise distances of chunks with length 10 s. Distance is defined as the length of the vector of maximal projections of the first 6 principal components.

The results presented in Fig. 11 corroborate the above hypothesis on the dimensionality of the behaviors. In particular, we observe the increase of the effective dimension if the chunk length is increasing, mixing different low dimensional behaviors. The latter point is made even more obvious in Fig. 12 depicting the overlap between the behaviors in chunks at different times. This overlap is large if the behaviors are essentially the same and small if the behavior has changed in the time span between the chunks. As the figure demonstrates, the overlap is indeed large for short time spans, but behaviors can reemerge after some time. Altogether, the results demonstrate that our TiPI maximization method effectively explores the behavior space of high-dimensional robotic systems by exciting their low-dimensional modes, avoiding in this way the curse of dimensionality.

## Discussion

Can a robot develop its skills completely on its own, driven by the sole objective to gain more and more information about its body and its interaction with the world? This question raises immediately further issues such as (i) what is the relevant information for the robot and (ii) how can one find a convenient update rule that realizes the gradient ascent on this information measure. We have studied the predictive information of the stream of sensor values as a tentative answer to the first question and, based on that, could give exact answers to the second question for simple cases. Earlier work was restricted to linear systems [14]. In order to be applicable to actual robotic systems we extend it to the case of nonlinear controllers and to nonstationary processes leading to a new measure called TiPI (time-local predictive information). Using several approximations we have been still able to obtained analytical results. In this way we derived an explicit exploration dynamics for the controller parameters based on an information maximization principle, namely by maximizing the TiPI using gradient ascent. For neural networks the gradient yields a fast synaptic dynamics which is essentially local in nature. Interestingly the TiPI landscape (on which the gradient is calculated) continuously changes its shape due to the general destabilization of the system dynamics inherent in maximizing the TiPI. For instance if the system dynamics is in an attractor, increasing the TiPI destabilizes the attractor until it may disappear altogether with a complete restructuring of the TiPI landscape. This is another reason why nonstationary processes have to be handled and why no convergence of the parameter dynamics is desired.

We studied a one-dimensional hysteresis system in order to work out the consequences of the nonstationary. The parameter dynamics leads to a slightly supercritical regime and additionally a self-induced hysteresis oscillation emerges. This is a useful new property as shown in the experiment with the Armband robot, a high-dimensional robot with a complicated dynamics. Despite the highly decentralized control–each joint is controlled individually–the robot develops coherent and global pattern of behavior. This is enabled by the continuous adaptation and spontaneous mutual cooperation of the individual controllers (hysteresis elements). We find the effect to be very robust against the speed of the exploration dynamics. Interestingly in the one-dimensional case the update formulas are independent of white noise and we can obtain an exploration dynamics in a fully deterministic system.

The new theoretical basis also allows for controlling complex high-dimensional robotic systems. This is demonstrated by a series of experiments with the Humanoid robot, now jointly controlled by a single high-dimensional controller. Given that there is no externally defined goal for the behavior development, will the robot develop a high behavioral variety depending on its physics and the environment it is dynamically embedded into? Our results support a positive answer to this question. We quantify the dimensionality and temporal structure of the behavior and find a succession of low-dimensional modes that increasingly explore the behavior space. Furthermore we show that environmental factors influence the internal as well as behavioral development. Without additional noise, the deterministic dynamics leads to an individual development which depends decisively on the particular experiences made during the lifetime.

The exploration dynamics can be viewed as a self-directed search process, where the directions to explore are created from the dynamics of the system itself. Without a random component the changes of the parameters are deterministically given as a function of the sensor values and internal parameters in a certain time window. For an embodied system this means in particular that constraints, responses and current knowledge of the dynamical interaction with the environment can directly be used to advance further exploration. Randomness is replaced with spontaneity which we demonstrate to restrict the search space automatically to the physically relevant dimensions. Its effectiveness is shown in the Humanoid experiments and we argue that this is a promising way to avoid the curse of dimensionality.

What is the relation of the parameter dynamics described here to other work on maximizing information quantities in neural systems? Maximizing the mutual information between input and output of a neuron, known as InfoMax, yields a very similar parameter dynamics [57]. Interestingly, when applied to a feed-forward network an independent component analysis can be performed. Also similar rules have been obtained in [58] where the entropy of the output of a neuron was maximized under the condition of a fixed average output firing-rate [58]. The resulting dynamics is called intrinsic plasticity as it acts on the membrane instead of on the synaptic level and it was shown to result in the emergence of complex dynamical phenomena [59]–[62]. In [63], [64] a related dynamics is obtained at the synaptic level of a feedback circuit realized by an autaptic (self) connection. In a recurrent network of such neurons it was shown that any finite update rate (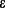 in our case) destroys all attractors, leading to intermittently bursting behavior and self-organized chaos.

Our work differs in two aspects. On the one hand, we use the information theoretical principle at the behavioral level of the whole system by maximizing the TiPI on the full sensorimotor loop, whereas they use it at the neuronal level. Nevertheless we manage to root the information paradigm back to the level of the synaptic dynamics of the involved neurons. On the other hand, as a direct consequence of that approach, there is no need to specify the average output activity of the neurons. Instead the latter is self-regulating by the closed loop setting. Independent of the specific realization, the general message is that these self-regulating neurons realize a specific working regime where they are both active and sensitive to influences of their environment. If embedded into a feedback setting many interesting phenomena are produced. Instead of studying them in internal (inside the “brain”) recurrences, we embed such neurons into a feedback loop with complex physical systems where the self-active and highly responsive nature of these neurons produces similar phenomena at the behavioral level.

In the current form, our approach is limited to the control of robots where the sensorimotor dynamics can be, in its essence, modeled by a simple feed-forward neural network. The parameter dynamics can also be calculated for more complex controllers, such as recurrent networks, which remains for future work. In this study only proprioceptive sensors measuring joint angles have been used. However, our newest experiences have shown that also other sensors e. g. current sensors, acceleration sensor or velocity sensors can be successfully integrated.

To conclude, information theory is a powerful tool to express principles to drive autonomous systems because it is domain invariant and allows for an intuitive interpretation. We present for the first time, to our knowledge, a method linking information theoretic quantities on the behavioral level (sensor values) to explicit dynamical rules on the internal level (synaptic weights) in a systematic way. This opens new horizons for the applicability of information theory to the sensorimotor loop and autonomous systems.

## Supporting Information

Text S1 **Appendix with derivations and technical detail.**
(PDF)

Video S1
**Armband robot starts to locomote and overcomes obstacles.** Each joint (hinge or slider) is individually and independently controlled by a one-dimensional TiPI maximizing controller. The locomotions starts due to spontaneous cooperation of the individual components and due to spontaneous symmetry breaking (going to left or right). The text in the video should say “Epsilon = 0.005”. Parameters: (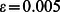, 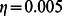).(MP4)

Video S2 **Humanoid robot on the ground.** One high-dimensional TiPI maximizing controller is used here (17 DoF). The controller starts from a small unit initialization, causing the robot to lay calmly. After an initial phase where the parameters adjust to create some activity we observe smooth patterns of behavior that patterns come and go with time. Within short time intervals one sees several repetitions of one mode until it vanishes and a new one emerges. Parameters: (epsilon = 0.0002, eta = 0.1).
(MP4)

Video S3 **Humanoid robot hanging at a bungee.** The bungee is not visualized. It acts as a spring force to the upper body. Its upper anchor point (visible as a yellow sphere at the end of the clip) not fixed in x-y, but only in its height, so the humanoid can in principle walk along the ground. See Video S2 for details. Note the different patterns of behavior just because of the different physical situation.
(MP4)

Video S4 **Humanoid robot at a high bar.** The hands of the robot are attached to a high-bar, however they remain free to rotate and move along the bar. See Video S2 for details. Note the different patterns of behavior just because of the different physical situation.
(MP4)

Video S5 **Humanoid robot falling into a pit.** See Video S2 for details. Note the different patterns of behavior just because of the different physical situation.
(MP4)
