# Supplementary material for: Information Driven Self-Organization of Complex Robotic Behaviors
Source: PLoS One. 2013 May 27;8(5):e63400. doi: 10.1371/journal.pone.0063400 (PMC3664628; doi:10.1371/journal.pone.0063400)
Supplement: Text S1 — Appendix with derivations and technical detail. (PDF) [file pone.0063400.s001.pdf]

Appendix **Text S1** for Information driven self-organization of complex robotic behaviors

Georg Martius Ralf Der Nihat Ay

A Estimating the TiPI

As stated above we consider the TiPI on the process of error propagations because it allows us to derive explicit expressions. Thus we start with the definition of the error propagation to derive eq. (11) and provide further insights.

As a first step, using the notion of an orbit of the dynamical system defined by the map $\psi : \mathbb{R}^n \to \mathbb{R}^n$ we define a sequence of states $\hat{s}_{t'} \in \mathbb{R}^n$

$$\hat{s}_{t'} = \psi^{t'-(t-\tau)}\left(s_{t-\tau}\right) \tag{A1}$$

for any time t' within the time window $t-\tau \le t' \le t$ starting from state $\hat{s}_{t-\tau} = s_{t-\tau}$. $\psi^k(s)$ denotes the k-fold iteration of the map ψ with $\psi^{(0)}\left(s\right) = s$. We can consider $\hat{s}_{t'}$ as the predicted state over $t' - (t-\tau)$ time steps. In particular, the prediction over τ steps is $\hat{s}_t = \psi^\tau\left(s_{t-\tau}\right)$.

The error propagation can now be defined as the difference

$$\delta s_{t'} = s_{t'} - \hat{s}_{t'} \tag{A2}$$

between the true state $s_{t'}$, eq. (6), and the state $\hat{s}_{t'}$ obtained by the deterministic dynamics (ψ), see Figure 1. The dynamics of the $\delta s_{t'}$ obeys the rule

$$\delta s_{t'} = L\left(s_{t'-1}\right)\delta s_{t'-1} + \xi_{t'} + O(\|\xi_{t'}\|^2) \tag{A3}$$

with starting state $\delta s_{t-\tau} = 0$ and $L(s)$ denoting the Jacobian matrix of ψ. This can be derived by using $\hat{s}_{t'} = \psi\left(\hat{s}_{t'-1}\right)$ and writing

$$\begin{aligned}\delta s_{t'} &= s_{t'} - \hat{s}_{t'} = \psi\left(s_{t'-1}\right) + \xi_{t'} - \psi\left(\hat{s}_{t'-1}\right)\\ &= \psi\left(\hat{s}_{t'-1} + \delta s_{t'-1}\right) - \psi\left(\hat{s}_{t'-1}\right) + \xi_{t'}\\ &= L\left(s_{t'-1}\right)\delta s_{t'-1} + \xi_{t'} + O(\|\xi_{t'}\|^2)\end{aligned}$$

In the following we will use this approximation which is arbitrary good for infinitesimally small noise. Note that this dynamics corresponds to that of a linear system, however with state dependent dynamical operator L. In a linear system, L is independent of the state and thus $\hat{s}_{t'} = L\hat{s}_{t'-1}$ such that the dynamical evolution of δs and s are the same.

As a remark, in the case of finite noise, we can obtain a related exact rule by using the mean value theorem of differential calculus stating that under mild restrictions one can find a state $\tilde{s}_{t'} \in [\hat{s}_{t'}, s_{t'}]$ so that

$$\delta s_{t'} = L\left(\tilde{s}_{t'-1}\right)\delta s_{t'-1} + \xi_{t'} \tag{A4}$$

yields the exact dynamics of the multi-step prediction error δs_t.

The interesting point now is that $I^\tau\left(S_t : S_{t-1}\right)$ (eq. (4)) is equal to that of the process defined by the error propagation dynamics, i. e.

$$I^\tau\left(S_t : S_{t-1}\right) = I^\tau\left(\delta S_t : \delta S_{t-1}\right) \tag{A5}$$

For the proof consider two random vectors S and S' together with the shifted vectors $U = S + a$ and $U' = S' + a'$. Using that the probability densities $p_S(s)$ and $p_U(u)$ obey $p_U(u) = p_U(s+a) = p_S(s)$ one obtains $H(S) = H(U)$. Analogously, the joint probability densities obey $p_{UU'}(u, u') = p_{UU}(s+a, s'+a') = p_{SS'}(s, s')$ so that $H(S'|S) = H(U'|U)$.

This result is central for the following arguments—we will make use of the fact that the dynamics eq. (A3) is more easily treated to obtain explicit estimates for the TiPI and its gradient.

Explicit expressions

By iterating eq. (A3) we obtain an explicit expression for δs_t (using here and in the following $L(t')$ for $L(s_{t'})$)

$$\delta s_t = \sum_{k=0}^{\tau-1} L^{(k)}(t-1)\, \xi_{t-k} \tag{A6}$$

with

$$L^{(k)}(t-1) = L(t-1) \cdots L(t-k)\,, \text{ and } L^{(0)} = \mathbf{I} \tag{A7}$$

for any t. In general it is very complicated to obtain the entropy of δS_t in realistic situations with high dimensional physical systems. Therefore we will base the further considerations on a convenient estimate of the latter. With white Gaussian noise, the process δS_t is Gaussian as well, i. e. $\delta S_t \sim \mathcal{N}(0, \Sigma_t)$ (it is a linear combination of independent Gaussians), so that the entropy is given in terms of the covariance matrix Σ_t of the random vector δS_t as [1]

$$H^{\tau}(\delta S_t) = \frac{1}{2} \ln |\Sigma_t| + \frac{n}{2} \ln 2\pi e \tag{A8}$$

$|A|$ denoting the determinant of a square matrix A and

$$\Sigma_t = \left\langle \delta S_t \delta S_t^{\top} \right\rangle = \int p(\delta s_t)\, \delta s_t \delta s_t^{\top}\, \mathrm{d}\delta s_t \tag{A9}$$

is the covariance matrix of δS_t and $p(\delta s_t)$ is the probability density distribution of the random variable δS_t. Using eq. (A6), explicit expressions for Σ can readily be obtained, see eq. (A13) below.

By the same arguments, the conditional entropy is defined, using eq. (7), as

$$H^{\tau}(\delta S_t | \delta S_{t-1}) = H^{\tau}(\Xi_t) = \frac{1}{2} \ln |D_t| + \frac{n}{2} \ln 2\pi e \tag{A10}$$

with

$$D_t = \left\langle \Xi_t \Xi_t^{\top} \right\rangle = \int p(\xi_t)\, \xi_t \xi_t^{\top}\, \mathrm{d}\xi_t \tag{A11}$$

where Ξ denotes the process of the noise with $p(\xi)$ being the probability density function of $\Xi \sim \mathcal{N}(0, D_t)$. Thus we obtain the estimate of the TiPI as

$$I^{\tau}(\delta S_t : \delta S_{t-1}) = \frac{1}{2} \ln |\Sigma_t| - \frac{1}{2} \ln |D_t| \tag{A12}$$

which is the entropy of the state δs minus that of the noise.

White noise

Explicit expressions revealing more details of the theory are obtained for the case of white noise, i. e. $\left\langle \xi_t \xi_{t'}^{\top} \right\rangle = \mathbf{0}$ if $t \neq t'$, so that using eq. (A6) in eq. (A9) yields

$$\Sigma = \sum_{k=0}^{\tau-1} L^{(k)} D \left(L^{(k)} \right)^{\top} \tag{A13}$$

In particular, in the case of $\tau = 2$, the shortest nontrivial time window, we find

$$\Sigma = D + LDL^{\top}.$$

It is also useful to introduce the transformed dynamical operator $\hat{L} = \sqrt{D^{-1}}L\sqrt{D}$ so that

$$\Sigma = \sum_{k=0}^{\tau-1} \sqrt{D}\hat{L}^{(k)} \left(\hat{L}^{(k)}\right)^{\top} \sqrt{D} \tag{A14}$$

and (using $\left|\sqrt{D}M\sqrt{D}\right| = |MD| = |M|\,|D|$)

$$I^{\tau}\left(\delta S_t : \delta S_{t-1}\right) = \frac{1}{2}\ln\left|\sum_{k=0}^{\tau-1} \hat{L}^{(k)} \left(\hat{L}^{(k)}\right)^{\top}\right| \tag{A15}$$

This corresponds to using a so-called whitening transformation on the state dynamics, replacing in eq. (A4) the state vector δs by a new vector $\delta x = \sqrt{D^{-1}}\delta s$ so that the covariance matrix of the noise in the δx dynamics is just the unit matrix.

Interestingly, the $\hat{L}$ operators also exist if the overall noise strength $\lambda = \|\xi\|$ goes to zero, so that I^{τ} stays finite although the defining entropies, conditioned on the state $s_{t-\tau}$, are equal to zero in the deterministic system. This can be seen by introducing $\hat{D} = \lambda^{-2}D$ where $\hat{D}$ stays finite with $\lambda \to 0$, we have $\hat{L} = \sqrt{\hat{D}^{-1}}L\sqrt{\hat{D}} = \sqrt{D}L\sqrt{D}$ since λ cancels out.

The linear case

For linear systems explicit expressions for the PI were obtained in [2]. In this case L is not dependent on the state s_t of the system so that $L^{(k)} = L^k$ in eq. (A7). Using eq. (A13), with $\tau \to \infty$, we reobtain the results of [2]. Note that all eigenvalues of the Jacobi matrix L must be less than one by absolute value so that the limes will exist. This requirement also guarantees that the conditioning on $s_{t-\tau}$ looses its influence for $\tau \to \infty$. Under the additional assumption that L is a normal matrix and the noise is isotropic the explicit expression $\Sigma = \left(\mathbb{I} - LL^{\top}\right)$ was obtained.

B Explicit gradient step

In order to derive the general gradient step on the TiPI based on eq. (13) we need to calculate the derivative $\frac{\partial}{\partial\theta}\ln|\Sigma_t|$. Considering any (square) matrix M depending on a single parameter θ_k of the set θ we have (see for example [3])

$$\frac{\partial}{\partial M}\ln|M| = \frac{1}{M^{\top}}$$

where we write $\frac{1}{M}$ for M^{-1} and

$$\frac{\partial}{\partial\theta_k}\ln|M| = \sum_{ij} M^{-1}_{ji}\frac{\partial M_{ij}}{\partial\theta_k} = \mathrm{Tr}\left(\left(M^{-1}\right)^{\top}\frac{\partial M}{\partial\theta_k}\right)$$

so that, using $\Sigma = \Sigma^{\top} = \left\langle \delta s \delta s^{\top}\right\rangle$ and omitting the time index

$$\frac{\partial}{\partial\theta_k}\ln|\Sigma| = \mathrm{Tr}\left(\frac{1}{\Sigma}\frac{\partial}{\partial\theta_k}\left\langle \delta s \delta s^{\top}\right\rangle\right) \tag{A16}$$

By using the cyclic invariance of the trace we obtain from eq. (A16)

$$\frac{\partial}{\partial\theta}\ln|\Sigma_t| = \left\langle \delta s_t^{\top}\Sigma^{-1}\frac{\partial}{\partial\theta}\delta s_t\right\rangle \tag{A17}$$

now valid for the entire set of parameters θ. By eq. (A4) we obtain (ignoring the dependence of ξ on the parameter)

$$\frac{\partial}{\partial\theta}\delta s_{t'}=\frac{\partial L\left(t'-1\right)}{\partial\theta}\delta s_{t'-1}+L\left(t'-1\right)\frac{\partial}{\partial\theta}\delta s_{t'-1}$$

so that by iteration

$$\frac{\partial}{\partial\theta}\delta s_{t}=\sum_{l=1}^{\tau-1}L^{(l-1)}\left(t-1\right)\frac{\partial L\left(t-l\right)}{\partial\theta}\delta s_{t-l}$$

where $L^{(k)}\left(t-1\right)$ is given in eq. (A7). Using $a^{\top}Wb=\left(W^{\top}a\right)^{\top}b$, we write

$$\frac{\partial}{\partial\theta}\ln\left|\Sigma_{t}\right|=\sum_{l=1}^{\tau-1}\left\langle \delta u_{t-l+1}^{\top}\frac{\partial L\left(t-l\right)}{\partial\theta}\delta s_{t-l}\right\rangle \tag{A18}$$

where (Σ is symmetric)

$$\delta u_{t-l+1}=\left(L^{(l-1)}\left(t-1\right)\right)^{\top}\Sigma_{t}^{-1}\delta s_{t} \tag{A19}$$

Stipulating the self-averaging property of the stochastic gradient, see section One-shot gradients for details, we realize the update rule as

$$\Delta\theta=\varepsilon\sum_{l=1}^{\tau-1}\delta u_{t-l+1}^{\top}\frac{\partial L\left(t-l\right)}{\partial\theta}\delta s_{t-l} \tag{A20}$$

Here we see again that $\tau=2$ is the simplest non-trivial case where the sum consists of a single term.

Characterizing the parameter dynamics

In order to better characterize the parameter dynamics, let us consider for the moment Σ at the r. h. s. of eq. (A16) to be some fixed, positive matrix (not depending on the parameters θ_k). Then, we can write

$$\mathrm{Tr}\left(\frac{1}{\Sigma}\frac{\partial}{\partial\theta_{k}}\left\langle \delta s\delta s^{\top}\right\rangle \right)=\frac{\partial}{\partial\theta_{k}}\left\langle \mathrm{Tr}\left(\frac{1}{\Sigma}\delta s\delta s^{\top}\right)\right\rangle =\frac{\partial}{\partial\theta_{k}}\left\langle \delta s^{\top}\frac{1}{\Sigma}\delta s\right\rangle$$

(using the cyclic invariance of the trace in the last step). The update rule eq. (13) becomes using again the self-averaging

$$\Delta\theta=\varepsilon\frac{\partial}{\partial\theta}\left\Vert \delta s\right\Vert _{\Sigma}^{2} \tag{A21}$$

where $\left\Vert a\right\Vert _{M}^{2}=a^{\top}M^{-1}a$ defines the length of a vector a in the metric given by M (considered fixed in the current gradient step). From eq. (A21) it becomes obvious that following the gradient is to increase the norm of δs in the Σ metric.

C Neural networks—derivation of the update rule

We derive the parameter dynamics for neural networks eq. (28) from the general parameter dynamics for the two-step time window given by eq. (14). According to eq. (26) we have $L=VG'\left(z\right)C+T$ with $z=Cs+h$ and $G'(z)=\mathrm{diag}[g_1'(z),\ldots,g_m'(z)]$. Putting this into

eq. (14) yields (omitting the time indices)

$$\begin{aligned}\frac{1}{\varepsilon}\Delta C_{ij} &= \delta u^{\top}\frac{\partial L}{\partial C_{ij}}\delta s \\ &= \delta u^{\top} V G' \frac{\partial C}{\partial C_{ij}}\delta s + \delta u^{\top} V \frac{\partial G'}{\partial C_{ij}} C\delta s \\ &= \left(G' V^{\top}\delta u\right)_i \delta s_j + \delta u^{\top} V \frac{\partial G'}{\partial C_{ij}} C\delta s \end{aligned} \tag{A22}$$

The second term remains to be calculated. Because G' is a diagonal matrix the vectors on both sides of the derivative carry the index i such that we get

$$\delta u^{\top} V \frac{\partial G'}{\partial C_{ij}} C\delta s = \left(\delta u^{\top} V\right)_i g_i''(z) s_j \left(C\delta s\right)_i \tag{A23}$$

In the case of $g\left(z\right) = \tanh\left(z\right)$ we find, using $g_i''\left(z\right) = -2g_i'\left(z\right) g_i\left(z\right)$ and $a = g(z)$

$$\delta u^{\top} V \frac{\partial G'}{\partial C_{ij}} C\delta s = -2\left(\delta u^{\top} V G'\right)_i \left(C\delta s\right)_i a_i s_j = -\gamma_i a_i s_j \tag{A24}$$

with

$$\gamma_i = 2\left(C\delta s\right)_i \delta\mu_i \tag{A25}$$

$$\delta\mu_i = \left(G' V^{\top}\delta u\right)_i \tag{A26}$$

The final update rule follows by putting eq. (A24) and eq. (A26) into eq. (A22)

$$\Delta C_{ij} = \varepsilon\delta\mu_i\delta s_j - \varepsilon\gamma_i a_i s_j \tag{A27}$$

Analogously we obtain the parameter dynamics of h as

$$\begin{aligned}\frac{1}{\varepsilon}\Delta h_i &= \delta u^{\top}\frac{\partial L}{\partial h_i}\delta s = \delta u^{\top} V \frac{\partial G'}{\partial h_i} C\delta s \\ &= \left(\delta u^{\top} V\right)_i g_i''(z)\left(C\delta s\right)_i = -\gamma_i a_i\end{aligned} \tag{A28}$$

A more compact matrix notation can be obtained by introducing the diagonal matrix Γ

$$\Gamma = \mathrm{diag}[\gamma_1, \cdots, \gamma_i]$$

and thus (reintroducing the time indices)

$$\frac{1}{\varepsilon}\Delta C_t = \delta\mu_t\delta s_{t-1}^{\top} - \Gamma_t a_t s_t^{\top} \tag{A29}$$

$$\frac{1}{\varepsilon}\Delta h_t = -\Gamma_t a_t \tag{A30}$$

In the case of arbitrary neuron activation functions g we obtain equivalent formula by defining

$$\gamma_i = -\frac{g_i''}{g_i' g_i}\left(C\delta s\right)_i \delta\mu_i \tag{A31}$$

Note the factor $-\frac{g_i''}{g_i' g_i}$ is 2 in the case of $g = \tanh$.

In the derivation of eqs. (A24) and (A28) we ignored the dependence of the state s in $g'\left(Cs + h\right)$ on the parameters C and h. This dependence can be considered explicitly if the state is at a fixed point. In that case, a more detailed discussion in [4] (section 6.2) shows that the effect of the derivative can be condensed into the so-called *sense* parameter α multiplying γ. Thus we replace γ as

$$\gamma_i \leftarrow \alpha\gamma_i \tag{A32}$$

where α is an empirical constant, typically $\alpha \geq 1$, by which the sensitivity of the sensorimotor dynamics to external perturbations can be regulated. This works also in more general cases like a limit cycle dynamics, see [4].

D Learning the inverse covariance matrix

Note that the covariance matrix given in eq. (15) can be easily obtained by the on-line update rule

$$\Delta\Sigma_t = \eta\left(\delta s_t \delta s_t^\top - \Sigma_t\right) \tag{A33}$$

or

$$\Sigma_{t+1} = (1-\eta)\,\Sigma_t + \eta\delta s_t \delta s_t^\top \tag{A34}$$

realizing a sampling over a restricted period of time. The update rate η defines the time horizon $t_H \propto \eta^{-1}$ for the averaging. The only remaining nontrivial operation in that setting is the inversion of the covariance matrix Σ. However, this can also be reduced to elementary operations by using the Sherman-Morrison formula as given by

$$\left(A + uv^\top\right)^{-1} = A^{-1} - \frac{1}{1 + v^\top A^{-1} u} A^{-1} u v^\top A^{-1}$$

Putting $A = (1-\eta)\,\Sigma$ and $uv^\top = \eta\delta s\delta s^\top$ we get

$$\left((1-\eta)\,\Sigma_t + \eta\delta s_t\delta s_t^\top\right)^{-1} = \frac{1}{1-\eta}\Sigma_t^{-1} - \frac{\eta}{(1-\eta)^2\left(1 + \frac{\eta}{1-\eta}\delta s_t^\top \Sigma_t^{-1}\delta s_t\right)}\Sigma_t^{-1}\delta s_t \delta s_t^\top \Sigma_t^{-1}$$

and thus

$$\Sigma_{t+1}^{-1} = \frac{1}{1-\eta}\Sigma_t^{-1} - \frac{\beta}{1-\eta}\Sigma_t^{-1}\delta s_t\delta s_t^\top\Sigma_t^{-1}$$

where $\beta \in \mathbb{R}$ is given by

$$\beta = \frac{\eta}{\left(1 - \eta + \eta\delta s_t^\top \Sigma_t^{-1}\delta s_t\right)}$$

Note that $\delta s_t^\top \Sigma_t^{-1}\delta s_t$ featuring in the denominator of β is a scalar so that with Σ_t^{-1} given there is no matrix inversion to be done.

If Σ_t is an $n \times n$ matrix, the cost of getting Σ_{t+1} is $O\left(n^2\right)$. This is very favorable if the dimension of the sensor space is large. Using the above formula, the only true inversion (of order $O\left(n^3\right)$) has to be done just once, when starting the process (with a convenient initialization of Σ).

References

1. Cover TM, Thomas JA (2006) Elements of Information Theory. Wiley.
2. Ay N, Bernigau H, Der R, Prokopenko M (2012) Information driven self-organization: The dynamical systems approach to autonomous robot behavior. Theory Biosci 131: 161-179.
3. Magnus J, Neudecker H (1988) Matrix differential calculus with applications in statistics and econometrics. New York, NY, USA: John Wiley & Sons.
4. Der R, Martius G (2012) The Playful Machine - Theoretical Foundation and Practical Realization of Self-Organizing Robots. Springer.
